# A supported psychoeducational intervention to improve family mental health following discharge from paediatric intensive care: feasibility and pilot randomised controlled trial

**DOI:** 10.1136/bmjopen-2015-009581

**Published:** 2015-12-24

**Authors:** Lorraine C Als, Simon Nadel, Mehrengise Cooper, Bea Vickers, M Elena Garralda

**Affiliations:** 1 Centre for Mental Health, Imperial College London, London, UK; 2 Department of Paediatric Intensive Care, St Mary's Hospital, Imperial College Healthcare NHS Trust, London, UK; 3 Department of Medicine, Imperial College London, London, UK; 4 Adolescent Assertive Outreach Team, South West London and St George's Mental Health NHS Trust, London, UK

**Keywords:** MENTAL HEALTH, Clinical trials < THERAPEUTICS

## Abstract

**Objective:**

To assess feasibility and pilot a supported psychoeducational tool to improve parent and child mental health following discharge from a paediatric intensive care unit (PICU), in preparation for a large randomised controlled trial (RCT).

**Design:**

Feasibility assessment and single-centre, parallel group, pilot RCT. A concealed computer generated list was used to randomise participants, with an allocation of 2:1 in favour of the intervention.

**Setting:**

A PICU in an acute care hospital in London, UK.

**Participants:**

31 parents of children aged 4–16 years-old admitted to PICU.

**Intervention:**

Parents received a psychoeducational tool supported by a telephone call. The psychoeducational tool outlined the possible psychological reactions in children and parents alongside management advice. The telephone call addressed each family's postdischarge experience, reinforced the psychoeducational material and encouraged parents to put into practice the advice given.

**Main outcome measures:**

The primary outcome was the number of feasibility criteria successfully met (linked to the intervention and the study design). Secondary outcomes were questionnaire data collected at 3–6-month follow-up assessing mental health in parents and children.

**Results:**

31 parents were randomised (intervention n=22; treatment as usual, TAU n=9). 23 parents were included in the analysis of secondary outcomes (intervention n=17; TAU n=6). 3 (of 6) intervention and 1 (of 6) study design feasibility criteria were fully met. All unmet criteria could be addressed with minor or significant modifications to the protocol. At follow-up there was a tendency for parents who received the intervention to report lower post-traumatic stress symptoms in themselves and fewer emotional and behavioural difficulties in their children than TAU parents. This needs to be explored in a fully powered trial.

**Conclusions:**

This feasibility and pilot RCT provided valuable information on the intervention and trial design for a full RCT.

**Trials registration number:**

NCT01737021; Results.

**Strengths and limitations of this study**

- There are few studies that have explored providing psychological support to families that have had a child admitted to paediatric intensive care unit (PICU). This study provided important insights into the feasibility and acceptability of the novel intervention and study design/procedures before conducting a full randomised controlled trial (RCT).
- The psychoeducational tool that formed part of the intervention was developed by expert and lay members including paediatricians, psychiatrists, psychologists and families with lived experience of having a child admitted to PICU.
- The intervention studied is innovative in its approach, in that it does not require families to return to the hospital. Such a strategy may potentially serve to increase the uptake of support in this difficult to reach population.
- This feasibility pilot RCT was performed at a single centre.
- The sample size fell short of its target.

## Introduction

It is becoming increasingly evident that PICU admission can have far-reaching psychological after effects including post-traumatic stress disorder (PTSD) in parents and children, parental anxiety and depression, and child emotional and behavioural problems.^1–6^ A recent review reported PTSD prevalence rates of 10–21% in parents and 5–28% in children following acute paediatric critical illness, with many other parents (up to 84%) suffering subclinical symptoms of PTSD, and with high correlations between parent and child PTSD symptoms.^7^

There are established associations between both parental mental health problems and parenting changes following critical illness and child mental health symptoms.^8^ For example, some mothers become more protective and strict, while others make more allowances for their child's behaviour. Successful interventions aimed at improving parental mental health and parenting may therefore be expected to have a beneficial effect on both parent and child mental health.

In 2009, NICE issued guidance on rehabilitation in adults after critical illness, recommending psychological follow-up for survivors and their family.^9^ However, there is currently no formal guidance in place for the follow-up of parents and their children after paediatric critical illness. There have been initiatives to evaluate different types of interventions. Melnyk *et al*,^10^ reported on the COPE programme, a three-phase preventative educational-behavioural intervention programme of audiotapes, written information and an activity workbook for parents and children to complete during and after the admission. They found some beneficial psychological effects over one-year follow-up. However, this was a multifaceted, comparatively complex and labour intensive intervention for young children (2–7 years old). The most significant beneficial findings were at the final 1 year follow-up, but they were subject to high attrition rates.

Other studies have involved less complex interventions, more in line with clinical practice, with offers of psychosocial parent follow-up to discuss any sequelae and provide support and guidance. These clinics have tended to be used by parents with mental health problems and the findings document small effect sizes in favour of the intervention for parental post-traumatic stress, anxiety and depression. However, uptake rates have been disappointing, ranging from 25% to 37%.^6^ ^11^ ^12^

We have developed an alternative intervention, offering psychoeducation and guidance to parents following their child's discharge from PICU by means of a carefully crafted written psychoeducational intervention tool supported by a follow-up telephone call. This aimed to increase accessibility of the intervention. Information-based interventions have been evaluated positively by parents and shown to be effective in reducing the parental stress associated with transfer from PICU to the general paediatric ward.^13^ ^14^ There are promising results in the use of such interventions following paediatric injury.^15^ ^16^ However, the impact of postdischarge psychoeducation on psychological sequelae in parents and their children following PICU admission has not been formally assessed. Screen and intervene approaches that include parental guidance on how to manage PTSD symptoms in children and psychosocial support for families have been recommended after childhood traumatic events.^17^ We therefore complemented the self-help psychoeducational tool with a supportive guidance telephone session.

The primary objective of this study was to assess the feasibility of the supported psychoeducational intervention tool and the design and procedures of an evaluative study. It was intended that the assessment of the process outcomes would feed directly into planning a full trial, providing information that would ultimately improve its operational aspects.^18^ ^19^ As secondary objectives, we aimed to obtain initial estimates of the effect of the intervention on parent and child mental health, and explore the moderating effect of baseline parental stress.^11^ ^20^ The study was not powered to assess statistical significance, and thus the analyses are mainly descriptive and should be interpreted with caution.^21–23^

## Methods

### Trial design

This was a single-centre, parallel group, RCT. Parents were individually randomised to either the intervention or treatment as usual (TAU) arm with an allocation ratio of 2:1 in favour of the intervention.

### Participants

Eligible participants were parents with a child aged 4–16 years old admitted to the PICU at St Mary's Hospital, London, UK, for at least 12 h. Exclusion criteria included child death prior to discharge; discharge to palliative care; planned admissions; history of prior PICU admission; overseas address; or insufficient English to complete study questionnaires. Parents were approached by PICU consultants prior to their child's discharge from PICU and invited to participate. If parents provided permission, once their child had been discharged home, they were then sent detailed information sheets and consent forms with instructions to complete them. All parents gave informed consent before taking part. If the child was aged 8 years or older, they provided assent to complete a self-report questionnaire at follow-up.

### Intervention

The intervention had two phases: the first phase, (ie, receipt of the psychoeducational tool), was planned to occur within 7 days of discharge from hospital and the second phase, (ie, receipt of the telephone call), within 14 days of receiving the tool.

The psychoeducational tool consisted of a handbook developed by mental health and paediatric experts and parents with lived experience of having a child in PICU. The handbook covered three main areas: emotional recovery, behavioural recovery and getting back to normal learning. The first section included a description of common emotional reactions in children, their siblings and parents following discharge from PICU, with advice regarding their management. It also included an outline of when recovery becomes stalled by the development of PTSD, its manifestations, what treatments are available and their rationale. The second section gave more detailed advice to parents about managing behavioural problems in children following hospital discharge. The third section addressed possible learning difficulties (eg, slowed information processing, memory and attention problems) in the aftermath of the child's admission and provided guidance on how to support affected children. There was an additional section containing a list of contacts of possible sources of further support and advice.

The telephone call, conducted by the researcher, was used to discuss each family's post-PICU experience, reinforce the material in the handbook (thus ensuring all families were exposed to the information), and support families in putting into practice the advice given, if appropriate.

### Outcomes

#### Primary outcome

There were 12 feasibility criteria used to judge the success of the trial (outlined in table 1). Six criteria related to the intervention (covering timings, compliance and evaluation) and six criteria related to the study design and procedures (covering screening, participation rate, acceptability of procedures, loss to follow-up and the time-scale of data collection). The following classification system was outlined for both the intervention and study design according to the number of criteria met: 0–2/6: not feasible/acceptable; 3–4/6: feasible/acceptable with modifications; 5/6: feasible/acceptable with close monitoring; 6/6: feasible/acceptable as it is.

**Table 1:** Feasibility objectives

Feasibility objectives	Questions	A priori criterion for success	Criterion met?	Outcome and contingency plans where appropriate
1. Feasibility/acceptability of intervention	Can the handbook be delivered within 7 days of hospital discharge?	A median time of 6 days	✗	The median time was 17 days (IQR: 11, 31.25) *>Consent/deliver tool in PICU*
	Can the telephone call be delivered within 14 days of phase 1?	A median time of 14 days	✗	The median time was 21 days (IQR: 14, 24) >*Change target to 3–6 weeks*
	Will parents read the handbook?	85% of parents will report reading the handbook	✓	All 17 (100%) responders said they had read the handbook
	Will it be possible to engage parents in the full intervention?	95% of parents will receive the full intervention	(✓)	18/22 (82%) parents could be engaged in the full intervention *>Rate reviewed as acceptable*
	Will parents evaluate the intervention as useful?	80% of parents will evaluate the intervention as useful	✓	All 17 (100%) responders evaluated the intervention as useful
	Will parents evaluate the intervention as appropriately timed?	80% of parents will deem timing of intervention as appropriate	✓	14/17 (82%) responders deemed the intervention as appropriately timed
2. Feasibility/acceptability of study design and procedures	How many families will be eligible to take part?	Mean of 5.3 eligible families are admitted to PICU per month	✗	The mean was four eligible families per month (range 1–8) *>Expand children's age range*
	What is the participation rate?	75% of eligible families agree to participate in the study	✗	31/59 (53%) of families agreed to participate *>Consent in PICU*
	Are families willing to be randomised?	Less than 10% non-participation rate due to randomisation procedures	✗	31% of non-participation due to prospect of randomisation *>Use patient and public involvement to improve explanation of research design*
	Is the loss to follow-up rate reasonable?	Less than 20% of families will fail to complete outcome measures	✗	Overall loss to follow-up was 8/22 (26%) >*Reduce the number of assessment measures*
	Can baseline data be collected in first week following discharge from hospital?	A median time from discharge to return of baseline questionnaires of 5 days	✗	The median time was 42 days (IQR: 35.5, 47.50) >*Baseline measures completed while on PICU*
	Can families be followed-up within 3–6 months of PICU discharge?	The median time from PICU discharge to follow-up is 5 months/150 days or less	✓	The median time was 150 days (IQR: 122, 180)

PICU, Paediatric Intensive Care Unit.

#### Secondary outcomes

Secondary outcomes included parent and child mental health after discharge from PICU, and exploration of the moderating effect of parental stress experienced during the PICU admission. Baseline and 3–6-month follow-up questionnaires were posted to families and returned using stamped addressed envelopes. We examined parental post-traumatic stress symptoms with the Impact of Events Scale (IES^24^) and anxiety and depression with the Hospital Anxiety and Depression Scale (HADS^25^). We assessed child emotional and behavioural difficulties with the parent-rated version of the Strength and Difficulties Questionnaire (SDQ^26^) and sleep with the parent-rated Child Sleep Habits Questionnaire (CSHQ^27^). For children aged 8–16 years, we assessed post-traumatic stress symptoms using the child-rated version of the IES-8.^28^

Parent recollections of stress during their child's PICU admission were measured using the Parental Stressor Scale: Paediatric Intensive Care Unit (PSS: PICU^29^). This questionnaire was completed retrospectively, once parents were back at home with their child.

### Sample size

Consistent with pilot studies, no power analysis was conducted. We aimed to recruit a minimum of 12 participants in the TAU group based on suggested guidelines for pilot studies.^30^

### Randomisation

Participants were randomised to the intervention or to TAU using a computer-generated list of random numbers prepared by an independent statistician. Randomisation was stratified by age (4–10 years and 11–16 years of age), with an allocation of 2:1 in favour of the intervention using random block sizes of 3 and 6. The allocation sequence was concealed from the researcher enrolling and assessing participants and was stored with an administrator who had no other involvement in the trial. After the researcher obtained the parent's consent and, if relevant, child's assent, they contacted the administrator for allocation consignment.

### Blinding

Owing to the nature of the trial, participants could not be blind to their allocation. There was one researcher recruiting, delivering the intervention, and assessing outcomes and thus it was not possible for them to be blind to intervention allocation.

### Analytical methods

The primary outcome was the number of feasibility criteria successfully met. Feasibility outcomes were assessed using descriptive statistics and evaluated according to the success criteria outlined in table 1. The number of criteria met was then assessed in line with the prespecified classification system.

The secondary outcomes included parent and child mental health. The initial plan was to assess changes in mental health outcomes from baseline to follow-up across both groups. However, as it did not prove feasible to collect baseline data within the specified time frame, we focused solely on the 3–6-month outcome data. Outcomes were assessed using total symptom scores. Descriptive data and effect sizes (Cohen's d) based on bootstrapped SDs of continuous data are reported. We intended to conduct supplementary analyses involving a 2 (stress: high stress vs low stress)×2 (group: intervention vs TAU) exploration of the role of parental stress on the efficacy of the intervention. However, this was precluded due to the small sample size.

## Results

### Participant flow

**Figure 1:**
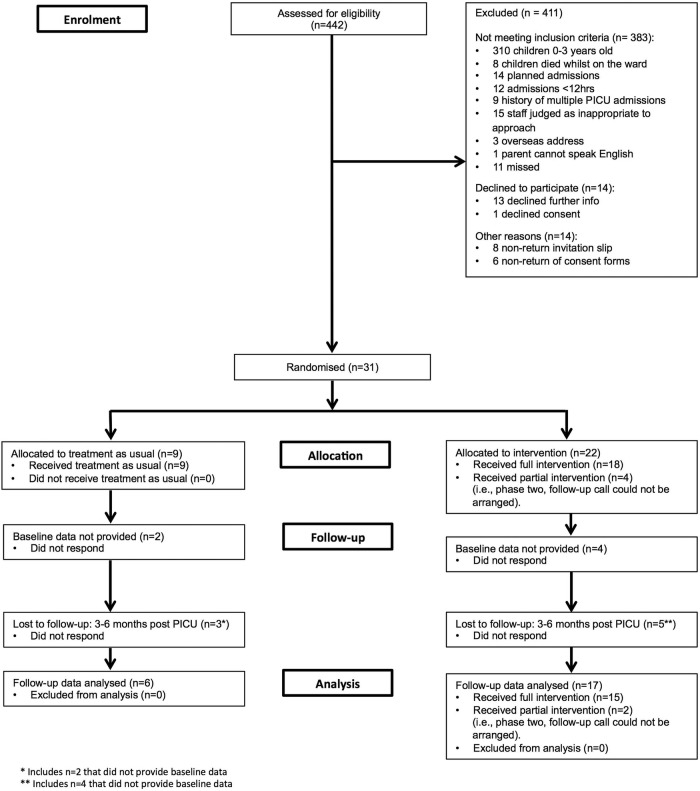
Participant flow chart. PICU,Paediatric Intensive Care Unit.

Figure 1 outlines the number of parent–child pairs randomly assigned, those receiving the intended treatment, losses and exclusions after randomisation, and those analysed (with reference to secondary outcome follow-up data).

### Recruitment

Eligible parent–child pairs were recruited from November 2012 to February 2014. Follow-up began in March 2013 and ended in July 2014. Families were approached for follow-up 3–6 months following their child's discharge from PICU: the median time from discharge to follow-up was 5 months (150 days; range 101–245 days).

### Numbers analysed

The mental health outcome data were assessed on an intention-to-treat basis and involved all parent–child pairs randomly assigned and providing follow-up data (17 in the intervention and 6 in TAU for parent reported data). Two parents in the intervention group were considered protocol violators as they did not receive the second phase of the intervention (ie, the telephone call), but they remained in the analyses as they provided follow-up data.

### Baseline data

Characteristics of the parents that provided data and their children, split by trial arm, are presented in table 2 and include age, gender, ethnicity, language, length of hospital stay, illness severity scores (PIM2^31^) and parental stress scores.

**Table 2:** Baseline demographic and clinic characteristics for families providing follow-up data in the intervention and treatment as usual groups

	n	Intervention group	n	Treatment as usual group
Parents				
Age, years	16	43.00 (42.00, 47.00)	6	36.00 (34.75, 41.00)
Fathers	17	4 (24%)	6	1 (17%)
White UK	16	7 (44%)	5	2 (40%)
English primary language	17	14 (82%)	6	4 (67%)
PSS: PICU score	17	3.13 (2.43, 3.64)	6	3.12 (2.88, 3.26)
Children				
Age, years	17	6.00 (5.50, 10.50)	6	9.00 (5.50, 11.00)
Male	17	7 (41%)	6	3 (50%)
White UK	16	5 (31%)	6	3 (50%)
Length of stay in PICU, days	17	5.00 (4.00, 12.50)	6	6.00 (4.00, 9.50)
Length of stay in hospital, days	15	10.00 (6.00, 21.00)	5	7.00 (3.50, 17.00)
PIM2, %	17	4.10 (1.20, 7.68)	6	6.69 (4.33, 16.33)

Data are presented as median (IQR) or frequency (%).

PIM2, Paediatric Index of Mortality 2; PSS: PICU, Parental Stressor Scale: Paediatric Intensive Care Unit.

### Outcomes and estimations

#### Primary outcomes

Met and unmet outcomes together with suggested modifications/protocol amendments are outlined in table 1.

Three out of six *intervention* feasibility and acceptability criteria were fully met: all parents said they had read the handbook, all evaluated it as useful, and most (82%) deemed it appropriately timed. Criteria not met included the time it took to execute both phases of the intervention, as well as the percentage of parents that engaged in the full intervention (although this was later reviewed as acceptable). Overall, the intervention was deemed feasible/acceptable with modifications.

In terms of the feasibility and acceptability of the *study design and procedures*, one criterion was fully met, namely families could be followed-up a median of 5 months post-PICU discharge. Criteria not met included the number of eligible families admitted to PICU per month, the participation rate, the refusal rate (due to randomisation), the number lost to follow-up and the time taken to return the baseline questionnaires. Thus, the study design and procedures were not deemed feasible/acceptable.

Additional comments collated from parents in the intervention group indicated that the information in the handbook made them feel more prepared for life after PICU (82%) and less anxious or concerned (77%). Almost half of the parents (47%) had shared the handbook with others including partners, relatives, their children (including the child admitted to PICU and their siblings) and teachers. With regards to the telephone call, 94% judged the timing to be good, 82% reported finding it useful and 59% thought that a single call was sufficient (35% were unsure about this).

#### Secondary outcomes

Parent and child mental health outcomes are outlined in table 3. Intervention parents reported fewer post-traumatic stress symptoms and depressive symptoms (small effect sizes), but there was little difference in anxiety scores (effect size <0.2). Table 3 shows that the children whose parents received the intervention had lower levels of total emotional and behavioural difficulties (moderate effect size), but there were negligible differences in sleep symptoms. Owing to questionnaire age-range criteria and missing data, the IES-8 data are based on reduced total numbers and thus we will not comment on this data.

**Table 3:** Mental health outcomes at 5 months post-PICU discharge for families providing follow-up data in the intervention and treatment as usual groups

	n	Intervention group	n	Treatment as usual group	Effect size d*
*Parent outcomes*					
Impact of Events Scale					
Post-traumatic symptoms total score	17	19.47 (11.64 to 26.62)	6	25.83 (11.47 to 39.00)	0.4
Hospital Anxiety and Depression Scale					
Anxiety total score	17	6.47 (4.53 to 8.54)	6	7.17 (4.20 to 11.00)	0.2
Depression total score	17	2.76 (1.33 to 4.45)	6	3.00 (0.00 to 5.96)	0.1
*Child outcomes*					
Strength and Difficulties Questionnaire					
Total Difficulties total score	14	9.21 (6.93 to 11.31)	6	11.83 (6.50 to 16.06)	0.6
Child Sleep Habits Questionnaire					
Sleep disturbance total score	13	47.08 (42.72 to 52.15)	6	48.00 (42.85 to 52.83)	0.1
Impact of Events Scale-8†					
Post-traumatic symptoms total score	3	13.00 (1.00 to 20.00)	3	8.33 (0.00 to 22.00)	–

Data are presented as means (BCa 95% CI) or frequency (%).

*Effect sizes for continuous data are based on boostrapped SD. An effect size between 0.2 and 0.5 being considered a small effect, 0.5 and 0.8 a moderate effect and 0.8 and above a large effect. An effect size was not calculated for the Impact of Events Scale-8 data due to reduced n.

†The Impact of Events Scale-8 was the only child self-report measure used and could only be completed by children aged 8–16 years-old, thus explaining the reduced ns.

## Discussion

We report the results of a combined feasibility and pilot RCT of a novel supported psychoeducational intervention to help parents of children admitted to PICU recognise and manage possible psychological sequelae in themselves and their children. The results confirm the acceptability and feasibility of many aspects of the intervention, with clear indications of modifications that could be made to improve on this further. Although the study design and procedures were not deemed feasible, the data we gathered provided sufficient information to guide significant protocol amendments in order to ensure the overall feasibility of a future full efficacy trial. The comparison of mental health outcomes in the intervention and TAU groups 5 months following PICU discharge show the intervention to hold promise for reducing mental health difficulties in parents and their children.

### 

#### Feasibility findings

Of the 12 feasibility criteria developed a priori for the study, 3 of 6 relating to the intervention and 1 of 6 addressing the study design and procedures were fully met. All unmet criteria were reviewed and appropriate modifications to the intervention were formulated as well as more significant amendments to the study design and procedures, leading us to conclude that the revised protocol would be acceptable and feasible for a larger study. Changes include (1) obtaining consent, collecting baseline data, randomising and delivering the psychoeducational tool while the child and their family are still on PICU (to help increase the participation rate, ensure baseline data are collected in a timely manner and that the psychoeducational tool is delivered promptly); (2) a delay of the supportive telephone call to 3–6 weeks after PICU discharge (in line with a time frame that was logistically viable and also considered acceptable by parents); (3) expanding the age range of children admitted into the study (to increase the number of eligible families); (4) reducing the number of assessment measures (to lessen the burden on participants and decrease the likelihood of attrition); and (5) working with patient and public involvement groups to provide a better explanation of the rationale for randomisation (to reduce non-participation on these grounds).

Notably, once parents were recruited to the study, it proved possible to provide the full intervention to 82%. This was initially considered an unmet criterion, as the target was 95%. On reflection, 82% was deemed acceptable, as this is a considerably higher rate than in previous studies offering outpatient consultations to families, where uptake in the intervention group ranged from 25% to 37%.^6^ ^11^ This suggests that providing after-care via a supported psychoeducational tool may be an effective way of increasing uptake of support in this difficult to reach population.

#### Secondary outcomes

Five months after PICU discharge, parents in the intervention group reported fewer post-traumatic stress symptoms and depressive symptoms than parents in the TAU group. Children of parents who received the intervention appeared to have fewer emotional and behavioural difficulties than those that received TAU. These differences were not subjected to statistical significance testing because our study was not powered to identify significant differences. Therefore, these findings need to be treated with caution and speculation about their meaningfulness is precluded. However, we believe the potential benefit of this supported psychoeducational intervention for parent and child mental health is worth exploring in a fully powered trial.

#### Strengths and limitations

Strengths of this study include its basis on empirical studies of PICU mental health outcomes, drawing and benefiting from the experience of previous well thought out, but ultimately unsuccessful intervention studies; the joint expert and lay approach to the development of the psychoeducational materials; the likely cost-effectiveness of the intervention; the careful approach to assessing feasibility and acceptability of both the intervention and study design/procedures. Limitations include falling short of the suggested minimum sample size for pilot studies; recruiting from a single centre, making generalisability uncertain; and the retrospective assessments of parental stress experienced while on PICU. As intended, however, the study opens the way for a future full RCT of the intervention.

## Conclusion

Our study indicates that our novel intervention, a psychoeducational tool supported by a directed telephone call, is acceptable to parents. Although aspects of the intervention and study design/procedures were not deemed feasible, we were able to address each unmet criteria, putting protocol modifications/amendments in place. In addition, preliminary results indicate the potential beneficial effects of this supported psychoeducational tool for the mental health of parents and children. However, this needs to be subjected to a fully powered study before this intervention can be widely introduced into clinical practice.
